# Development and validation of a nomogram to predict the prognosis of patients with squamous cell carcinoma of the bladder

**DOI:** 10.1042/BSR20193459

**Published:** 2019-12-23

**Authors:** Mei-Di Hu, Si-Hai Chen, Yuan Liu, Ling-Hua Jia

**Affiliations:** 1 Department of General Practice, The First Affiliated Hospital of Nanchang University, Nanchang 330006, China; 2 Department of Gastroenterology, The First Affiliated Hospital of Nanchang University, Nanchang 330006, China; 3 Division of Nephrology, The Fifth People’s Hospital of Shanghai, Fudan University, Shanghai 200240, China; 4 Department of Urology, Jiangxi Provincial People’s Hospital Affiliated to Nanchang University, Nanchang 330006, China

**Keywords:** Decision curve analysis, Nomogram, Prognosis, Squamous cell carcinoma of the bladder, TNM stage

## Abstract

**Background:** The present study aimed to develop and validate a nomogram based on expanded TNM staging to predict the prognosis for patients with squamous cell carcinoma of the bladder (SCCB).

**Methods:** A total of 595 eligible patients with SCCB identified in the Surveillance, Epidemiology, and End Results (SEER) dataset were randomly divided into training set (*n* = 416) and validation set (*n* = 179). The likelihood ratio test was used to select potentially relevant factors for developing the nomogram. The performance of the nomogram was validated on the training and validation sets using a C-index with 95% confidence interval (95% CI) and calibration curve, and was further compared with TNM staging system.

**Results:** The nomogram included six factors: age, T stage, N stage, M stage, the method of surgery and tumor size. The C-indexes of the nomogram were 0.768 (0.741–0.795) and 0.717 (0.671–0.763) in the training and validation sets, respectively, which were higher than the TNM staging system with C-indexes of 0.580 (0.543–0.617) and 0.540 (0.484–0.596) in the training and validation sets, respectively. Furthermore, the decision curve analysis (DCA) proved that the nomogram provided superior clinical effectiveness.

**Conclusions:** We developed a nomogram that help predict individualized prognosis for patients with SCCB.

## Introduction

Urinary bladder cancer is the seventh common cancer among men and the seventeenth among women worldwide [1]. Urinary bladder cancer has several subtypes such as squamous cell carcinoma (SCC), urothelial carcinoma (UC) and adenocarcinoma. SCC of the bladder (SCCB) could be subdivided into Schistosoma related and non-Schistosoma related tumors, with the latter being the most common subtype in the developed countries [2]. Patients with SCCB are generally diagnosed at late stage and have very poor prognosis [3]. Currently, SCCB patients are mainly treated with radical cystectomy (RC). The evaluation of the prognosis of SCCB patients after treatment is mainly based on American Joint Committee for Cancer (AJCC) [4]. Due to the various factors that may affect cancer progression, the evaluation of cancer prognosis based on AJCC staging alone is unpredictable [5]. Therefore, there is an urgent need to develop new methods to accurately predict the prognosis and improve the management of SCCB patients.

Recently, a nomogram has been shown as a reliable model for the perdition of prognosis of cancer patients [6]. A nomogram is a graphical illustration of a mathematical model, in which different factors are pooled together to predict a definite endpoint, and has been utilized as a convenient and reliable tool to predict the outcome of cancer patients [7]. Unfortunately, there is still no nomogram has been reported to predict the prognosis of SCCB patients. Therefore, in the present study we aimed to develop and validate a nomogram to predict the survival of SCCB patients based on the population-based data from the Surveillance, Epidemiology, and End Results (SEER) database including AJCC TNM staging system.

## Materials and methods

### Patient eligibility and study variables

**Figure 1:**
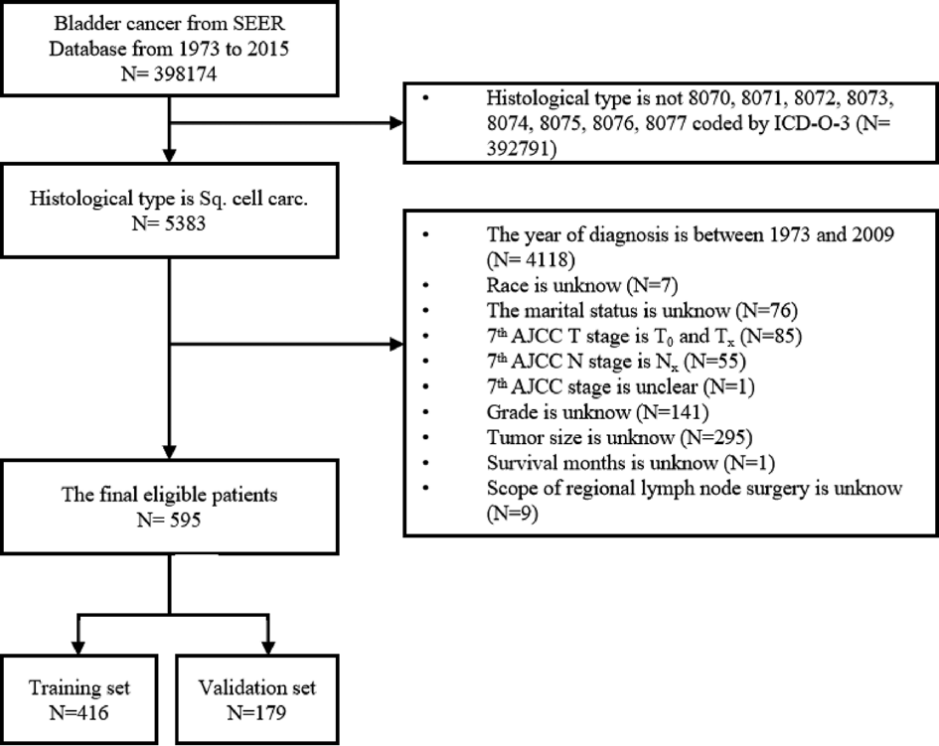
The flow diagram of selection of eligible patients

The present study was performed at the First Affiliated Hospital of Nanchang University and Jiangxi Provincial People’s Hospital. Ethics requirement was not required because of no direct involvement with human participants or animals. Patients diagnosed with SCCB from 1973 to 2015 were selected from SEER database. Patients were included if they fulfilled the diagnosis of bladder cancer, and the histological type was confirmed as SCCB (8070-8077) based on International Classification of Diseases for Oncology (ICD-O-3). Patients were excluded if unclear and incomplete information were recorded. The detailed flow chart for patient selection was shown in Figure 1. Total 15 variables were selected in the present study, including the age, race, gender, year of diagnosis, marital status, histologic type based on the International Classification of Diseases for Oncology (3rd Edition codes), American Joint Committee on Cancer (7th edition) stage I-IV, AJCC T stage, AJCC N stage, AJCC M stage, surgery of primary site, scope of regional lymph node surgery, tumor size, survival months and status. To properly evaluate the prognostic value of tumor size in SCCB patients, we identified 45 and 96 mm as the cut-off point for patients by Х-tile, a professional tool for cut-off point decision [8].

### Statistical analysis

The present study enrolled 595 patients who were randomly divided into training set (*n* = 416) and validation set (*n* = 179). A chi-square test was employed to compare the differences in demographics and tumor characteristics between the training and validation sets. Univariate and multivariate Cox proportional hazards regression analyses were used to evaluate prognostic variables in the training set in order to develop the nomogram. Furthermore, the independent prognostic factors were selected to develop the nomogram.

### Construction and validation of the nomogram

A nomogram was constructed when variables were selected from above steps. The performence of the nomogram in the training and validation was calculated by concordance-index (C-index), which is similar to the area under curve (AUC) of the receiver operating characteristic (ROC) curve. If the C-index is 0.60, the nomogram will discern a patient that will die from a patient that will not die at success rate of 60% [9]. Univariate and multivariate Cox proportional hazards regression analyses were performed using the SPSS version 24.0 software (IBM Corporation, U.S.A.). A calibration plot was used to assess the deviation between the predicted and actual probabilities. The calculation of the C-index and the construction of the nomogram and calibration plot were processed with the R statistical package “rms”, “survival” and “foreign” (R software version 3.5.2). Additionally, the decision curve analysis (DCA) was performed using the source file “stdca.r*”*, which was downloaded from decisioncurveanalysis.org. Two-sided *P* value below 0.05 was considered to be statistically significant.

## Results

### Patient characteristics

The present study enrolled 595 eligible patients with SCCB between 2010 and 2015, who were randomly divided into training set (*n* = 416) and validation set (*n* = 179). The patient characteristics are listed in Table 1. The high incidence of age ranged between 50 and 69 years. Total 477 (80.2%) patients had non-lymphatic metastasis, and 542 (91.1%) patients were in M0.

**Table 1:** Characteristics of patients

	All patients (*n* = 595)	Training set (*n* = 416)	Validation set (*n* = 179)	*P* value
**Age (year), *n* (%)**				0.036
≤49	56 (9.4)	47 (11.3)	9 (5.0)	
50–69	220 (37.0)	143 (34.4)	77 (43.0)	
70–79	150 (25.2)	103 (24.8)	47 (26.3)	
≥80	169 (28.4)	123 (29.6)	46 (25.7)	
**Race, *n* (%)**				0.285
Black	69 (11.6)	53 (12.7)	16 (8.9)	
White	504 (84.7)	346 (83.2)	158 (88.3)	
Other^1^	22 (3.7)	17 (4.1)	5 (2.8)	
**Sex, *n* (%)**				0.172
Male	288 (48.4)	209 (50.2)	79 (44.1)	
Female	307 (51.6)	207 (49.8)	100 (55.9)	
**Marital status, *n* (%)**				0.756
Married	275 (46.2)	194 (46.6)	81 (45.3)	
Unmarried	320 (53.8)	222 (53.4)	98 (54.7)	
**AJCC stage, *n* (%)**				0.150
I^2^	70 (11.8)	56 (13.5)	14 (7.8)	
II	178 (29.9)	116 (27.9)	62 (34.6)	
III	173 (29.1)	122 (29.3)	51 (28.5)	
IV	174 (29.2)	122 (29.3)	52 (29.1)	
**AJCC T, *n* (%)**				0.041
T1	78 (13.1)	64 (15.4)	14 (7.8)	
T2	206 (34.6)	137 (32.9)	69 (38.5)	
T3	175 (29.4)	116 (27.9)	59 (33.0)	
T4	136 (22.9)	99 (23.8)	37 (20.7)	
**AJCC N, *n* (%)**				0.737
Non-lymphatic metastasis	477 (80.2)	335 (80.5)	142 (79.3)	
Lymphatic metastasis	118 (19.8)	81 (19.5)	37 (20.7)	
**AJCC M, *n* (%)**				
M0	542 (91.1)	376 (90.4)	116 (92.7)	
M1	53 (8.9)	40 (9.6)	13 (7.3)	
**Grade, *n* (%)**				0.885
I	58 (9.7)	41 (9.9)	17 (9.5)	
II	230 (38.7)	159 (38.2)	71 (39.7)	
III	217 (36.5)	150 (36.1)	67 (37.4)	
IV	90 (15.1)	66 (15.9)	24 (13.4)	
**Surgery of primary site, *n* (%)**				0.663
None	345 (58.0)	241 (57.9)	104 (58.1)	
Local excision	227 (38.2)	157 (37.7)	70 (39.1)	
Surgery	23 (3.9)	18 (4.3)	5 (2.8)	
**Scope of regional lymph node surgery, *n* (%)**				0.809
None, Biopsy	297 (49.9)	209 (50.2)	88 (49.2)	
≥1 regional lymph nodes	298 (50.1)	207 (49.8)	91 (50.8)	
**Tumor size (mm), *n* (%)**				0.368
≤45	186 (31.3)	136 (32.7)	50 (27.9)	
46–96	333 (56.0)	225 (54.1)	108 (60.3)	
≥97	76 (12.8)	55 (13.2)	21 (11.7)	

**Note:** other^1^ comprises American Indian/Alaska Native, Asian/Pacific Islander.

I^2^ comprises AJCCstage Oa, Ois, I.

### Prognostic factors in the training set

To identify the prognostic factors, we performed the univariate and multivariate cox regression analyses. The results are listed in Table 2. By univariate analysis eight variables (age, marital status, AJCC T stage, AJCC N stage, AJCC M stage, surgery of primary site, scope of regional lymph node surgery and tumour size) were significant risk factors for survival. By multivariate analysis six variables were identified as independent prognostic factors, including age, AJCC T stage, AJCC N stage, AJCC M stage, surgery of primary site and tumor size.

**Table 2:** Univariate and multivariate analysis of the training set

	Univariate analysis		Multivariate analysis	
	HR (95%CI)	*P* value	HR (95%CI)	*P* value
**Age (year)**				
≤49	Reference		Reference	
50–69	0.852 (0.542–1.338)	0.485	1.080 (0.673–1.736)	0.749
70–79	1.125 (0.708–1.788)	0.619	1.705 (1.036–2.804)	0.036
≥80	1.972 (1.270–3.064)	0.003	2.556 (1.561–4.188)	<0.001
**Race**				
Black	Reference			
White	0.849 (0.603–1.194)	0.346		
Other^1^	0.599 (0.290–1.237)	0.166		
**Sex**				
Male	Reference			
Female	1.267 (0.993–1.616)	0.057		
**Marital status**				
Married	Reference		Reference	
Unmarried	1.290 (1.010–1.649)	0.042	1.046 (0.808–1.354)	0.734
**AJCC T**				
T1	Reference		Reference	
T2	0.907 (0.619–1.332)	0.619	1.567 (1.046–2.346)	0.029
T3	0.650 (0.432–0.979)	0.039	1.961 (1.190–3.231)	0.008
T4	1.721 (1.717–2.531)	0.006	3.249 (2.009–5.254)	<0.001
**AJCC N**				
Non-lymphatic metastasis	Reference		Reference	
Lymphatic metastasis	1.785 (1.348–2.365)	<0.001	1.567 (1.124–2.186)	0.008
**AJCC M**				
M0	Reference		Reference	
M1	3.718 (2.615–5.287)	<0.001	2.747 (1.857–4.064)	<0.001
**Grade**				
I	Reference			
II	0.867 (0.554–1.357)	0.533		
III	1.072 (0.687–1.674)	0.759		
IV	0.953 (0.577–1.573)	0.849		
**Surgery of primary site**	2.457 (2.007–3.009)	<0.001		
None	Reference		Reference	
Local excision	2.413 (1.874–3.107)	<0.001	2.324 (1.431–3.776)	0.001
Surgery	6.266 (3.784–10.374)	<0.001	3.804 (1.953–7.407)	<0.001
**Scope of regional lymph node surgery**				
None, Biopsy	Reference		Reference	
≥1 regional lymph nodes	0.404 (0.315–0.519)	<0.001	0.719 (0.447–1.157)	0.174
**Tumor size (mm)**				
≤45	Reference		Reference	
46–96	2.105 (1.565–2.829)	<0.001	1.817 (1.340–2.463)	<0.001
≥97	3.587 (2.425–5.307)	<0.001	2.405 (1.587–3.646)	<0.001

**Note:** other^1^ comprises American Indian/Alaska Native, Asian/Pacific Islander.

### The development of nomogram

**Figure 2:**
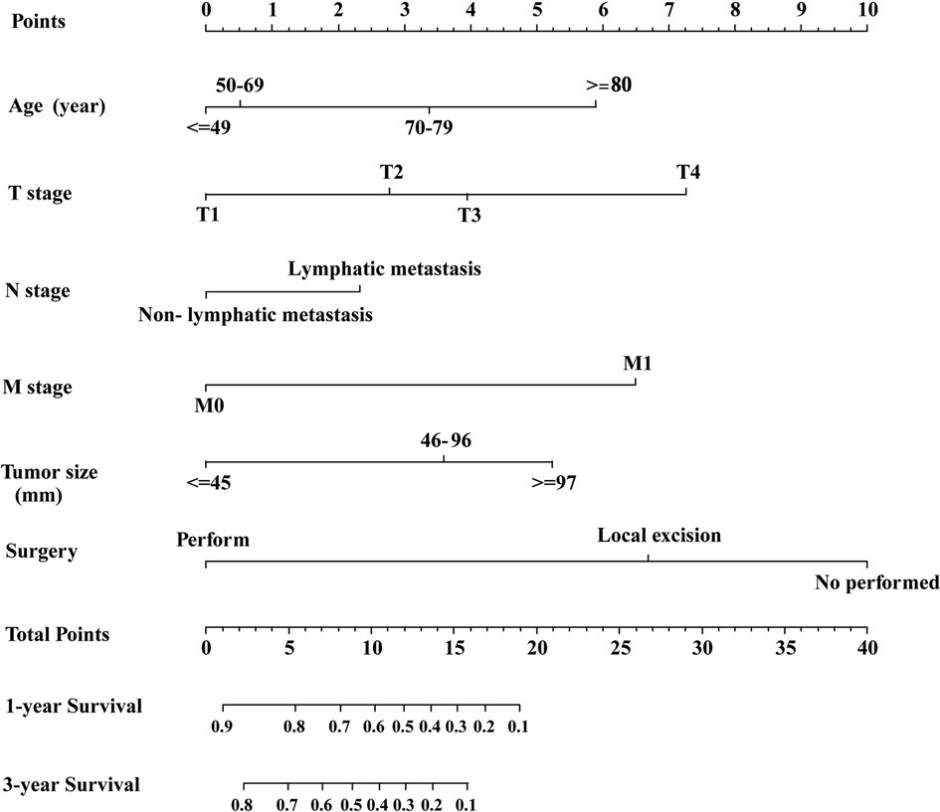
The nomogram for predicting 1- and 3-year survival of SCCB patients

Based on the analysis of prognostic factor in multivariate analysis, six variables (age, AJCC T stage, AJCC N stage, AJCC M stage, surgery of primary site and tumor size) were selected to develop the nomogram for predicting 1- and 3-year survival rates (Figure 2). Each variable was assigned a score ranging from 0 to 10 on a point scale. By calculating the total score of various covariates and placing the total score on a total point scale, the 1- and 3-year survival rates could be efficiently estimated for a patient. The points of all variables are listed in Table 3. The nomogram model demonstrated that surgery method had the largest contribution to survival rate, followed by AJCC T, AJCC M, age, tumour size and AJCC N.

**Table 3:** Points of all variables in nomogram

Variable	Nomogram score
**Age (year)**	
≤49	0
50–69	1
70–79	3
≥80	6
**AJCC T**	
T1	0
T2	3
T3	4
T4	7
**AJCC N**	
Non-lymphatic metastasis	0
Lymphatic metastasis	2
**AJCC M**	
M0	0
M1	6
**Tumor size (mm)**	
≤45	0
46-96	4
≥97	5
**Surgery**	
Performed	0
Local excision	7
No performed	10

### The validation of nomogram

**Figure 3:**
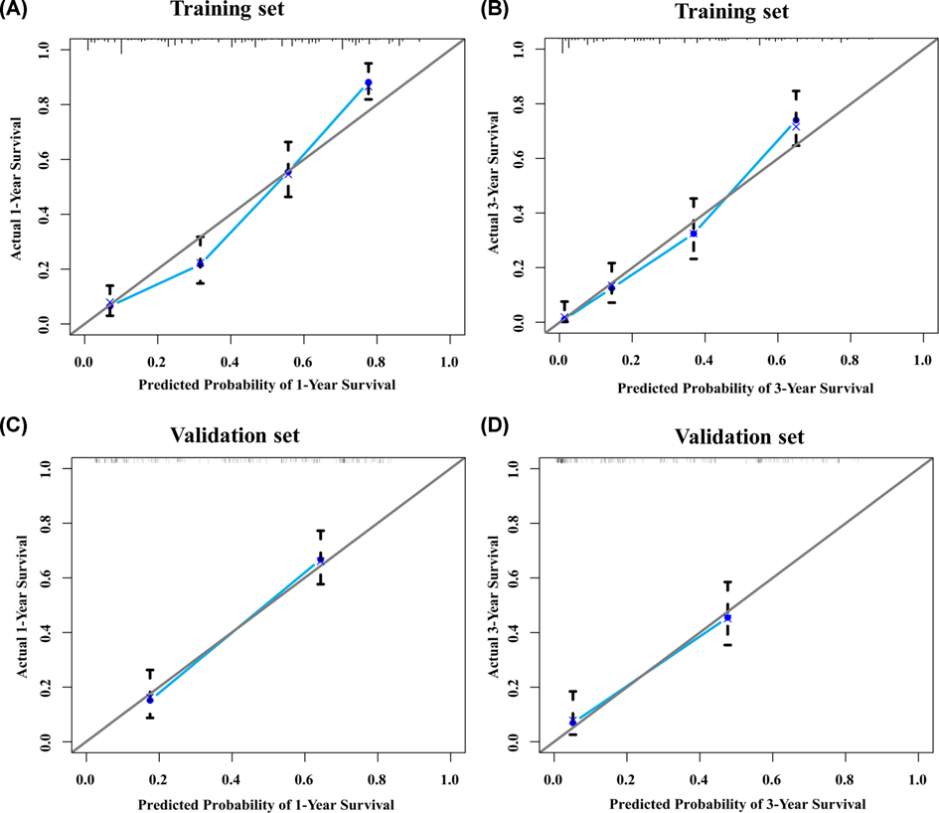
Calibration plot of the nomogram (**A**) 1-year survival nomogram calibration curves in training set. (**B**) 3-year survival nomogram calibration curves in training set. (**C**) 1-year survival nomogram calibration curves in validation set. (**D**) 3-year survival nomogram calibration curves in validation set.

**Figure 4:**
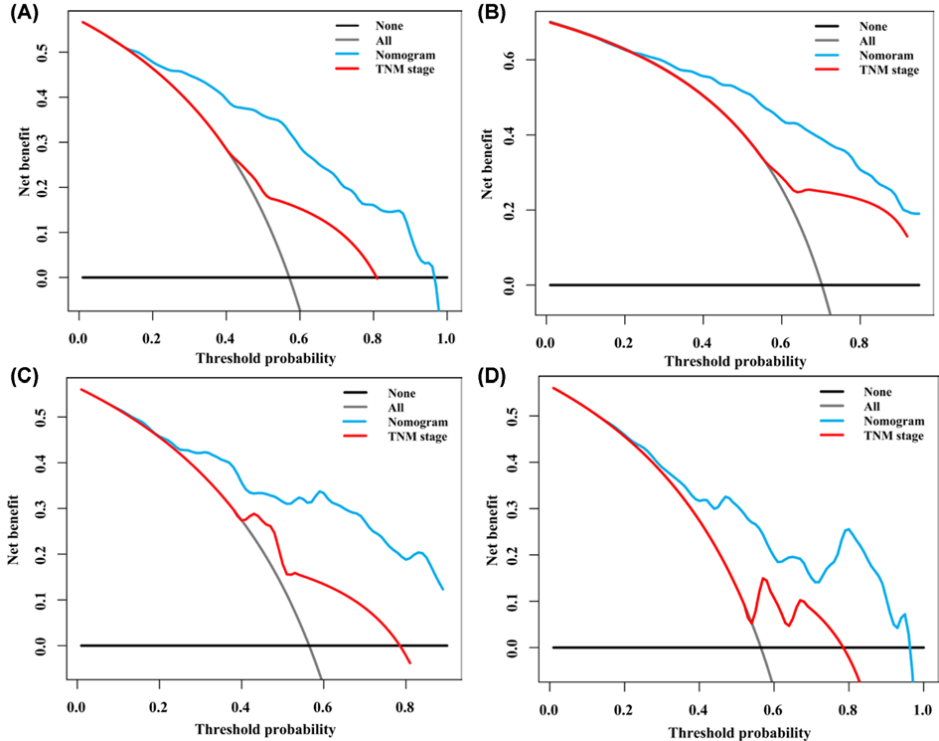
DCA of the nomogram and AJCC TNM stage for predicting survival of SCCB patients (**A**) 1-year DCA in training set. (**B**) 3-year DCA in training set. (**C**) 1-year DCA in validation set and (**D**) 3-year DCA in validation set.

The C-indexes of the nomogram for the prediction of overall survival in the training and validation sets were 0.768 (95%CI: 0.741–0.795) and 0.717 (95%CI: 0.671–0.763), respectively. The C-indexes for the TNM staging system both in training set 0.580 (95%CI: 0.543–0.617) and in validation set 0.540 (95%CI: 0.484–0.596) were significantly lower than the nomogram system. Calibration plots were made to examine the accuracy of nomogram for the predicting of 1- and 3-year survival rates and the results showed that the accuracy was good (Figure 3). Decision curve analysis (DCA) of the nomogram indicated that the nomogram had a wide and practical range of threshold probability for the training and validation sets for predicting 1- or 3- year survival rates (Figure 4). Furthermore, the nomogram had broader range of threshold probability and higher net benefits than AJCC TNM staging system.

## Discussion

Because most SCCB patients are diagnosed at late stage and their prognosis is poor, it is important to develop a valuable system to predict the prognosis of these patients. Currently, the 7th AJCC TNM staging system is good for the staging of SCCB patients, but it could not effectively predict the survival of the patients [10]. In the present study, we developed a comprehensive nomogram model based on SEER to better predict the prognosis of SCCB patients. C-index, calibration plot and DCA curve were performed to evaluate the discrimination, calibration and clinic utility of the nomogram, respectively, and the results showed that the nomogram was better than the TNM staging system to predict the 1- and 3-year survival of SCCB patients.

To our knowledge, this is the first nomogram that integrated personalized characteristics, AJCC TNM staging, tumor characteristics, and the treatment method to predict the prognosis of SCCB. Although our nomogram was based on AJCC TNM staging, the C-index of this nomogram was higher than that of AJCC TNM staging system in both training and validation sets. Therefore, this nomogram showed improved power of discrimination. In addition, the C-index of this nomogram exceeded 0.7 for both training and validation sets, indicating the adequate power of discrimination [11]. DCA showed that this nomogram provided superior clinical utility.

Recently, several nomograms incorporating a variety of variables have been developed for predicting the prognosis of patients with different cancers [12–15]. In particular, Tang et al. developed nomograms to predict overall survival and cancer-specific survival in patients with T1 high-grade bladder cancer. Unfortunately, their model is only applicable to T1 high-grade bladder cancer [16]. Xu et al. developed and validated a nomogram based on radiomics and clinical predictors for the prediction of recurrence risk of bladder cancer [17]. Their results are based on relatively small number of patients from a single center and need further validation. Interestingly, a recent study validated a nomogram based on European multicenter prospective cohort to predict the mortality after radical cystectomy in a Japanese cohort, and reported good results [18]. In the present study, our nomogram contained 6 variables, including 1 personalized variable (age), 3 variables based on the AJCC TNM staging system, 1 surgical variable (surgical method) and 1 variable about the primary site of the SCCB (tumor size). All 6 variables included in this nomogram could be obtained easily, which facilitates the application of this nomogram in clinical practice. Among the variables included in this nomogram, the method used to treat the primary site was the most important prognostic factor. Therefore, clinicians could use this nomogram to predict 1- and 3- year survival rates of SCCB patients. For example, for a 65-year-old patient presents with a 50-mm tumor, with T2 stage and without lymphatic metastasis or distant metastasis, if this patients undergoes surgery, the 1- and 3-year survival rates will both be over 50% according to our nomogram. However, if the patient refuses surgery, the 1- and 3-year survival rates will be below 20%.

Several limitations of the present study should be pointed out. First, this nomogram was based on the 7th AJCC TNM staging system, and need further optimization based on the 8th AJCC TNM staging system [19]. Second, this nomogram only selected limited variables. Whether increasing variables will improve this nomogram need further study. However, the complexity of the nomogram could increase accordingly and may diminish clinical utility. Third, the use of nomograms has its own drawbacks. For example, nomogram assumed that the overall mortality of SCCB patients had been static during 2010 to 2015.

In summary, we developed a nomogram integrating TNM staging and other clinical variables to predict 1- and 3-year survival rates for SCCB patients. Based on the validation of discrimination, probability calibration and decision curve analysis, we demonstrated that this nomogram had adequate power of discrimination and satisfactory calibration. Compared with the 7th AJCC staging system, this nomogram is user-friendly and accurate for prognostic prediction for SCCB patients. This nomogram could be a promising tool to predict the prognosis of SCCB patients.

## Availability of data and materials

All data are included within the article.

## Abbreviations

AJCC: American Joint Committee for Cancer
DCA: decision curve analysis
RC: radical cystectomy
SCC: squamous cell carcinoma
SCCB: squamous cell carcinoma of the bladder
UC: urothelial carcinoma
